# The Effects of Immunosuppressive Treatment during Pregnancy on the Levels of Potassium, Iron, Chromium, Zinc, Aluminum, Sodium and Molybdenum in Hard Tissues of Female Rats and Their Offspring

**DOI:** 10.3390/ijms21239038

**Published:** 2020-11-27

**Authors:** Daniel Styburski, Wojciech Żwierełło, Marta Skórka-Majewicz, Marta Goschorska, Irena Baranowska-Bosiacka, Joanna Kabat-Koperska, Dariusz Chlubek, Izabela Gutowska

**Affiliations:** 1 Department of Medical Chemistry, Pomeranian Medical University in Szczecin, Powstancow Wlkp Street 72, Szczecin 70-111, Poland; daniel.styburski@interia.pl (D.S.); wojciech.zwierello@gmail.com (W.Ż.); marta_skorka@o2.pl (M.S.-M.); 2 Department of Biochemistry and Medical Chemistry, Pomeranian Medical University in Szczecin, Powstancow Wlkp Street 72, Szczecin 70-111, Poland; rcmarta@wp.pl (M.G.); ika@pum.edu.pl (I.B.-B.); dchlubek@pum.edu.pl (D.C.); 3 Department of Nephrology, Transplantology and Internal Medicine, Pomeranian Medical University in Szczecin, Powstancow Wlkp 72, Szczecin 70-111, Poland; joanna.koperska@pum.edu.pl

**Keywords:** immunosuppressive therapy, pregnancy, elements content, bone, tooth

## Abstract

The ideal immunosuppressive regimen should provide for excellent immunosuppression with no side effects. Yet, current immunosuppressive therapy regimens commonly used in clinical applications fail to meet this criterion. One of the complications caused by immunosuppressive drugs is mineralization disorders in hard tissues. In this study, we evaluated the effects of three immunosuppressive therapies used after transplantation on the levels of potassium, iron, chromium, zinc, aluminum, sodium and molybdenum in the bones and teeth of female rats and their offspring. The study was conducted on 32 female Wistar rats, subjected to immunosuppressive regimens (cyclosporine A, mycophenolate mofetil and prednisone; tacrolimus, mycophenolate mofetil and prednisone; and cyclosporine A, everolimus and prednisone). The hard tissues of rats were analyzed using inductively coupled plasma optical emission spectrometry (ICP-OES, ICAP 7400 Duo, Thermo Scientific) equipped with a concentric nebulizer and a cyclonic spray chamber. All the immunosuppressive regimens included in the study affected the concentrations of the studied minerals in hard tissues of female rats and their offspring. The therapy based on cyclosporine A, everolimus and prednisone led to a decline in the levels of iron in bone, zinc in teeth, and molybdenum in the bone and teeth of mothers, while in the offspring, it caused a decline of bone potassium, with a decrease in iron and increase of molybdenum in teeth. Moreover, the regimen caused an increase in aluminum and chromium in the teeth and aluminum in the bones of the offspring, and consequently, it seems to be the therapy with the most negative impact on the mineral metabolism in hard tissues.

## 1. Introduction

Successful organ transplantation is made possible thanks to the suppression of the body’s natural immune reactions, which would otherwise lead to transplant rejection. The function of the immune system can be altered through the administration of immunosuppressive agents. Medications most commonly used in immunosuppressive regimens include corticosteroids (prednisone), calcineurin inhibitors (CNI) (cyclosporine A (CsA) and tacrolimus (Tc)) and mycophenolate mofetil (MMF) [1]. They are, however, well known to have numerous side effects, notably including loss of bone mass, with the most pronounced decrease observed in the first six months post-transplantation and occurring mainly in cortical bone. In the course of subsequent months, the rate of bone loss slows down, chiefly due to lower corticosteroid use [2].

Immunosuppressive therapy is also a necessity in pregnant transplant recipients, in whom the medications as well as their active metabolites, passing through the placenta, may exert adverse effects on the developing fetus. Immunosuppressive drugs and their metabolites cause chromosomal aberrations, structural defects, organ-specific toxicity and intrauterine growth retardation, increasing the risk of prematurity and spontaneous abortion [3,4]. It is believed that two years make for a safe interval between organ transplantation and conception, as by that time, the pre-emptive antiviral therapy has been completed, and the levels of immunosuppressive medication are low. Some studies, however, propose three years as the safe interval [5].

A typical regimen used in immunosuppressive therapy for pregnant transplant recipients includes a CNI (Tc or CsA), azathioprine (AP) and prednisone. On the other hand, due to the strong teratogenic effects, mycophenolate mofetil (MMF) and sirolimus (rapamycin) should be avoided in these patients [4].

Bone is an active tissue that undergoes continuous remodeling, with coordinated interactions between osteoclasts involved in bone resorption and osteoblasts responsible for new bone formation and mineralization [6]. Apart from the nutrients used as bone-building material, normal bone remodeling requires an adequate supply of macro- and micro-elements to regulate bone metabolism as needed. Their positive effects on the health of hard tissues are increasingly recognized; hence in recent years, there has been a growing interest in research investigating the role of minerals in normal bone remodeling [6,7]. Seen as immunosuppressive medications may disrupt hard tissue mineralization [2], in this study, we have decided to evaluate the effects of three post-transplantation immunosuppressive therapies, featuring medications which are indicated (prednisone, CsA, Tc) and contraindicated (MMF, everolimus) during pregnancy. The study regimes are currently the most widely used to suppress the immune response in transplant patients [4]. The components of the regimens are designed to complement each other through different mechanisms of lowering immunity; therefore, the components of the regimens were glucocorticosteroids, mTor inhibitors, CNI, IMPDH inhibitors. As a result, it is possible to reduce doses of the drug while maintaining the therapeutic effect [1,3,4]. We sought to find out whether the type of immunosuppressive therapy impacts the levels of analyzed minerals (potassium, iron, chromium, zinc, aluminum, sodium and molybdenum) in the hard tissues of pregnant rats and their offspring and also whether the immunosuppressive regimens included in the study may pose a threat to mineral metabolism in the bones and teeth of the mother and fetus.

## 2. Results

### 2.1. Effects of Immunosuppressive Therapy on the Levels of Studied Minerals (K, Fe, Cr, Zn, Al, Mo) in Rat Bone Tissue

Mother rats subjected to immunosuppressive treatment.

**Figure 1:**
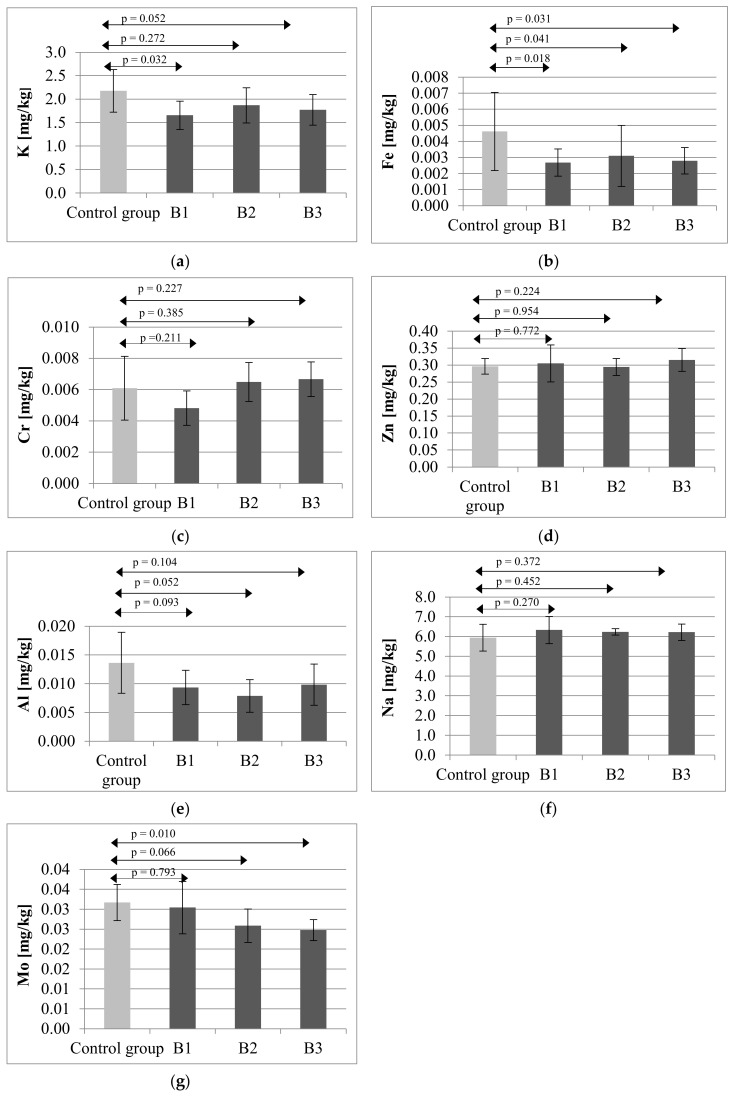
Mean concentration and SD of potassium (**a**), iron (**b**), chromium (**c**), zinc (**d**), aluminum (**e**), sodium (**f**) and molybdenum (**g**) in bones of rat’s mothers after immunosuppressive therapy. B1—rat’s mothers receiving cyclosporine (CsA) (5 mg/kg/day), mycophenolate mofetil (MMF) (20 mg/kg/day), prednisone (4 mg/kg/day); B2—rat’s mothers receiving tacrolimus (Tc) (4 mg/kg/day), MMF (20 mg/kg/day), prednisone (4 mg/kg/day); B3—rat’s mothers receiving CsA (5 mg/kg/day), everolimus (0.5 mg/kg/day), prednisone (4 mg/kg/day). Statistically significant differences—*p* ≤ 0.05.

The mean potassium level in bone in the control group amounted to 2.18 mg/kg (SD = 0.45). In terms of the mean potassium levels in the bone of the mother rats subjected to immunosuppressive therapies, the lowest mean K level was observed in group B1, exposed to CsA, MMF, prednisone (1.66 mg/kg; SD = 0.30), which constituted a significant difference (*p* = 0.032) compared to the control group (Figure 1a).

The highest mean bone concentration of iron in mothers was observed in the control group (0.0046 mg/kg; SD = 0.0024). In group B1, the mean Fe level amounted to 0.0027 mg/kg (SD = 0.0008), in group B2:0.0031 mg/kg (SD = 0.0019), and in group B3:0.0028 mg/kg (SD = 0.0008). Statistical analysis confirmed that the differences in Fe concentrations between the control group and each of the study groups were significant: B1 (*p* = 0.018), B2 (*p* = 0.041), B3 (*p* = 0.031) (Figure 1b).

Chromium levels in the bones of mother rats were similar in the control group (0.0061 mg/kg; SD = 0.0020), group B2 and group B3, whereas the Cr level in group B1 amounted to 0.0048 mg/kg; (SD = 0.0011). The statistical analysis did not confirm the significance of the differences between the control and experimental groups B1 (*p* = 0.211), B2 (*p* = 0.385), B3 (*p* = 0.227) (Figure 1c).

Zinc content was similar across all the groups. In the case of this mineral, no statistically significant differences were found between the analyzed groups B1 (*p* = 0.772), B2 (*p* = 0.954), B3 (*p* = 0.224) (Figure 1d).

The highest mean level of aluminum in rat bone tissue was observed in the control group (0.0136 mg/kg; SD = 0.0053), but statistical analysis did not confirm the significance of the differences between the control and experimental groups B1 (*p* = 0.093), B2 (*p* = 0.052), B3 (*p* = 0.104) (Figure 1e).

Samples were also analyzed for sodium content, which was similar across all the groups. In this case, too, no statistically significant differences were found between the analyzed groups B1 (*p* = 0.270), B2 (*p* = 0.452), B3 (*p* = 0.372) (Figure 1f).

The highest mean level of molybdenum was observed in the control group (0.0317 mg/kg; SD = 0.0046). A similar result was noted in group B. However, markedly lower levels of Mo were observed in group B2 receiving Tc, MMF and prednisone (0.0259 mg/kg; SD = 0.0042) and in group B3 receiving CsA, everolimus and prednisone (0.0248 mg/kg; SD = 0.0026). Statistical analysis revealed a significant difference between the control and group B3 (*p* = 0.011) (Figure 1g).

### 2.2. Offspring of Mothers Receiving Immunosuppressive Treatment

In the second part of the study, bones of the offspring of female rats receiving immunosuppressive treatment were analyzed for mineral content.

**Figure 2:**
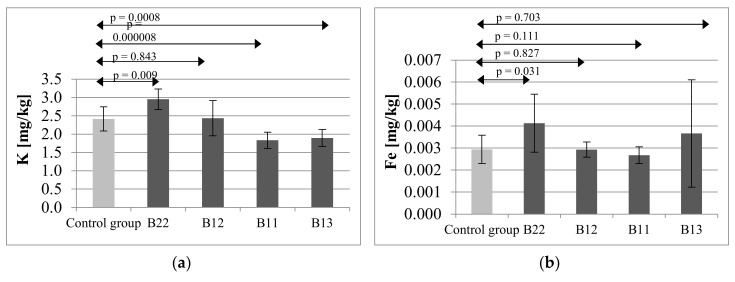

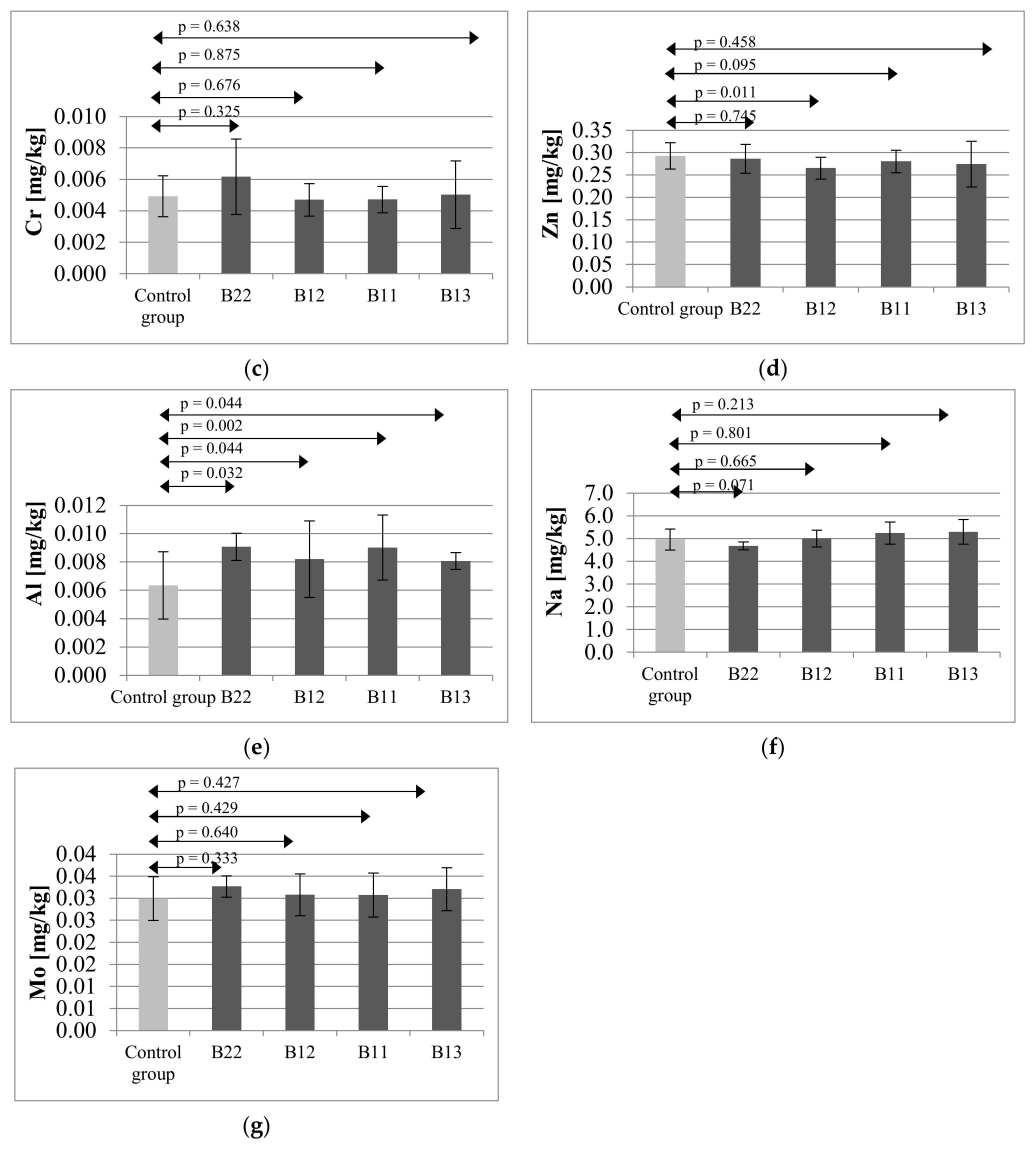
Mean concentration and SD of potassium (**a**), iron (**b**), chromium (**c**), zinc (**d**), aluminum (**e**), sodium (**f**) and molybdenum (**g**) in bones of offspring of rat’s mothers receiving immunosuppressive therapy. B22—offspring of rat’s mothers receiving CsA (5 mg/kg/day), MMF (20 mg/kg/day), prednisone (4 mg/kg/day); B12—offspring of rat’s mothers receiving Tc (2 mg/kg/day), MMF (10 mg/kg/day), prednisone (4 mg/kg/day); B11—rat’s mothers receiving CsA (2.5 mg/kg/day), MMF (10 mg/kg/day), prednisone (4 mg/kg/day); B13—rat’s mothers receiving CsA (2.5 mg/kg/day), everolimus (0.25 mg/kg/day), prednisone (4 mg/kg/day). Statistically significant differences—*p* ≤ 0.05.

The highest mean potassium level was observed in the group exposed to full doses of CsA, MMF and prednisone (group B22), amounting to 2.9525 mg/kg (SD = 0.2827), while in the offspring of mothers receiving half-doses of the same immunosuppressive agents (group B11), the mean level amounted to 1.8333 mg/kg (SD = 0.2233). In the offspring group exposed to Tc, MMF and prednisone (group B12), the mean potassium content in bone amounted to 2.4366 mg/kg (SD = 0.4782). A similar level of the mineral was observed in the control group (2.4163 mg/kg; SD = 0.3309). The potassium level in the offspring group exposed to CsA, everolimus and prednisone (group B13) amounted to 1.8931 mg/kg (SD = 0.2334). Statistical analysis revealed significant differences between the control and groups B22 (*p* = 0.009), B11 (*p* = 0.000008) and B13 (*p* = 0.0008) (Figure 2a).

The highest iron level in bone tissue of the offspring of mothers receiving immunosuppressants was found in group B22 (0.0041 mg/kg; SD = 0.0013) and was significantly higher than the level in the control group 0.0029 mg/kg (SD = 0.0006) (*p* = 0.031) (Figure 2, b).

The mean content of chromium in bone of the offspring in the control group amounted to 0.0049 mg/kg (SD = 0.0013), in group B22:0.0062 mg/kg (SD = 0.0024), in group B12:0.0047 mg/kg (SD = 0.0010), in group B11:0.0047 mg/kg (SD = 0.0008), and in group B13:0.0050 mg/kg (SD = 0.0022). In the case of this mineral, statistical analysis did not reveal statistically significant differences B22 (*p* = 0.325), B12 (*p* = 0.676), B11 (*p* = 0.875), B13 (*p* = 0.638) (Figure 2c).

Zinc content was similar in the control group (0.2929 mg/kg; SD = 0.0295), group B22, group B11 and in group B13, whereas in the group of offspring exposed to Tc, MMF and prednisone (group B12; 0.2654 mg/kg; SD = 0.0243) it was significantly lower (*p* = 0.011) (Figure 2d).

In the assessment of aluminum content in the bones of the offspring exposed to immunosuppressive treatment, the lowest level of this mineral was observed in the control group (0.0063 mg/kg; SD = 0.0024). In group B22, the Al level amounted to: 0.0091 mg/kg (SD = 0.0010), in group B12:0.0082 mg/kg (SD = 0.0027), in group B11:0.0090 mg/kg (SD = 0.0023), and in group B13:0.0081 mg/kg (SD = 0.0006). Statistical analysis revealed significant differences between the control and study groups: B22 (*p* = 0.032), B12 (*p* = 0.045), B11 (*p* = 0.002), B13 (*p* = 0.044) (Figure 2e).

The lowest mean concentration of sodium was observed in group B22—offspring exposed to full doses of CsA, MMF and prednisone. In the control group, the Na level amounted to 4.9653 (SD = 0.4622), and in group B12:5.0052 mg/kg (SD = 0.3702). The highest Na levels were observed in group B11 (5.2443 mg/kg; SD = 0.4863) and group B13 (5.2971 mg/kg; SD = 0.5458), but statistical analysis did not reveal significant differences between the groups B22 (*p* = 0.071), B12 (*p* = 0.665), B11 (*p* = 0.801), B13 (*p* = 0.213) (Figure 2f).

The mean content of Mo in the femoral bone of the offspring of mother rats exposed to immunosuppressive therapies in the control group amounted to 0.0300 mg/kg (SD = 0.0049). The highest mean concentration of molybdenum was observed in group B22 and amounted to 0.0327 mg/kg (SD = 0.0024). In the case of this mineral, no statistically significant differences were identified, either B22 (*p* = 0.333), B12 (*p* = 0.640), B11 (*p* = 0.429), B13 (*p* = 0.427) (Figure 2g).

### 2.3. Effects of Immunosuppressive Therapy on the Levels of Studied Minerals (K, Fe, Cr, Zn, Al, Mo) in Rat Teeth

Mother rats subjected to immunosuppressive treatment.

**Figure 3:**
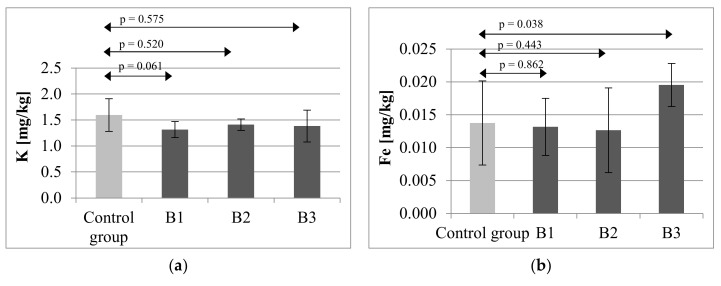

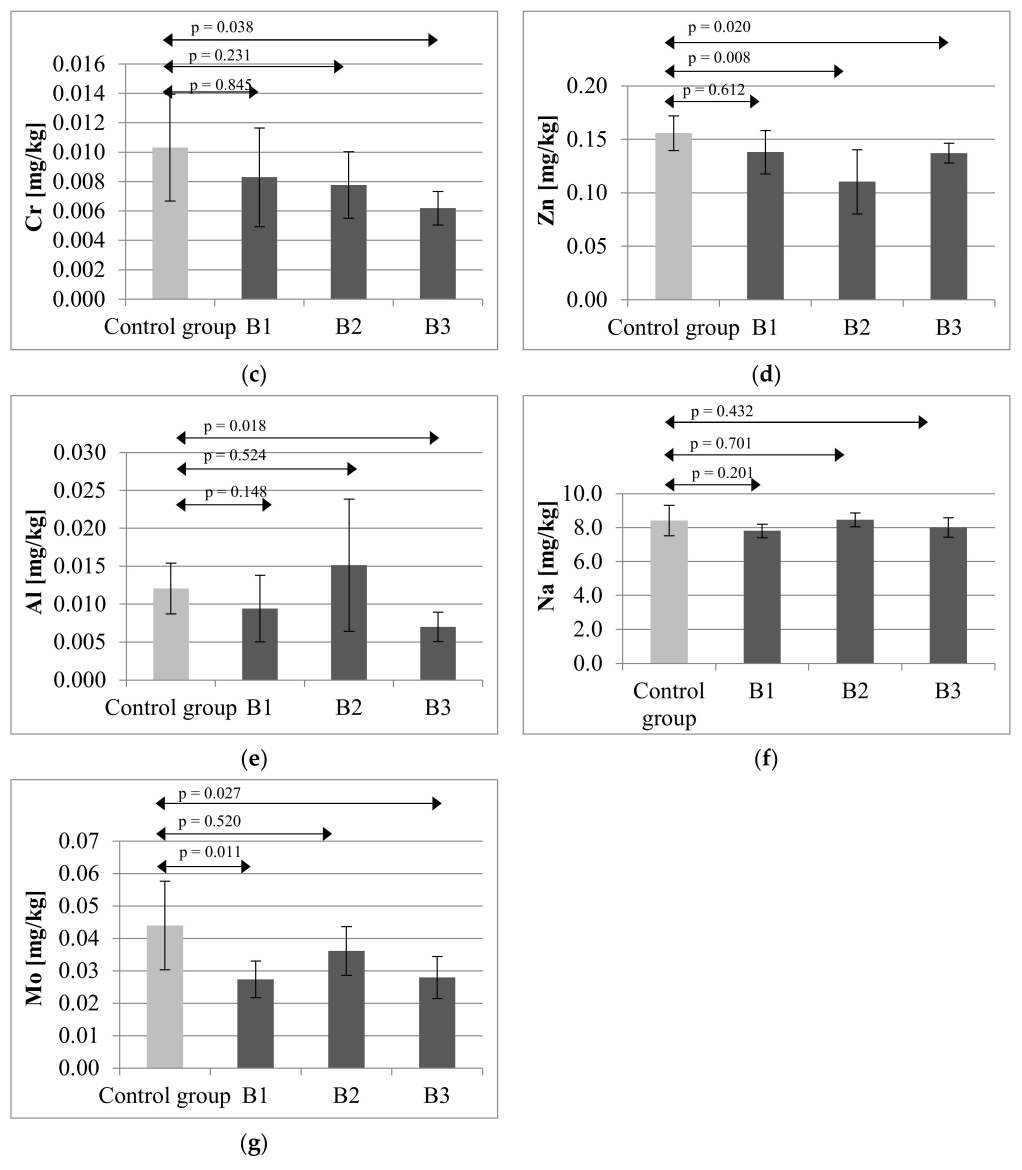
Mean concentration and SD of potassium (**a**), iron (**b**), chromium (**c**), zinc (**d**), aluminum (**e**), sodium (**f**) and molybdenum (**g**) in teeth of rat’s mothers after immunosuppressive therapy. B1—rat’s mothers receiving CsA (5 mg/kg/day), MMF (20 mg/kg/day), prednisone (4 mg/kg/day); B2—rat’s mothers receiving Tc (4 mg/kg/day), MMF (20 mg/kg/day), prednisone (4 mg/kg/day); B3—rat’s mothers receiving CsA (5 mg/kg/day), everolimus (0.5 mg/kg/day), prednisone (4 mg/kg/day). Statistically significant differences—*p* ≤ 0.05.

Analysis of dental tissues of the mothers receiving immunosuppressive therapies showed that the highest mean level of K, amounting to 1.5955 mg/kg (SD = 0.3133) was observed in the control group, while the lowest level: 1.3154 mg/kg (SD = 0.1545) was noted in group B1, receiving CsA, MMF and prednisone. Potassium levels were comparable across all the studied groups, hence statistical analysis failed to identify any significant differences B1 (*p* = 0.061), B2 (*p* = 0.520), B3 (*p* = 0.575) (Figure 3a).

The highest mean concentration of iron in the teeth of mother rats was observed in group B3 (0.0195 mg/kg; SD = 0.0033). In group B1, the mean Fe level amounted to 0.0132 mg/kg (SD = 0.0043), in group B2:0.0126 mg/kg SD = 0.0064, and in the control group: 0.0138 mg/kg (SD = 0.0064). Statistical analysis confirmed statistically significant differences in Fe concentrations in teeth between the control group and B3 (*p* = 0.038) (Figure 3b).

The highest level of chromium was observed in the control group (0.0103 mg/kg; SD = 0.0036), whereas the lowest level of the mineral was observed in group B3 (0.0062 mg/kg; SD = 0.0011). Statistical analysis confirmed statistically significant differences in Cr concentrations in teeth between the control group and B3 (*p* = 0.038) (Figure 3c).

The highest Zn level was observed in the control group (0.1558 mg/kg; SD = 0.0163), and the lowest in group B2 (0.1103 mg/kg; SD = 0.0301). Statistical analysis revealed a significant difference between the control and group B2 (*p* = 0.008221). Similar levels of the mineral were observed in groups B1 (0.1381 mg/kg; SD = 0.0203) and B3 (0.1372 mg/kg; SD = 0.0093), but because of the high standard deviation statistical significance was confirmed only between the control and B3 (*p* = 0.020) (Figure 3d).

Analysis of dental tissue for aluminum content revealed that the lowest level of the mineral was found in group B3 (0.0070 mg/kg; SD = 0.0019). This result was significantly lower than that in the control group, amounting to 0.0121 mg/kg (SD = 0.0033) (*p* = 0.018) (Figure 3e).

Sodium content in the control group and all the studied group was on similar levels; therefore, no statistically significant differences between the analyzed groups were identified for this mineral. B1 (*p* = 0.201), B2 (*p* = 0.701), B3 (*p* = 0.432) (Figure 3f).

The mean level of molybdenum among mothers receiving immunosuppressive therapies in the control group amounted to 0.0440 mg/kg (SD = 0.0136). The lowest levels determined in groups B1 (0.0274 mg/kg; SD = 0.0056) and B3 (0.0280 mg/kg; SD = 0.0065). Statistical analysis revealed significant differences between the control and groups B1 (*p* = 0.011) and B3 (*p* = 0.027) (Figure 3g).

### 2.4. Offspring of Mothers Receiving Immunosuppressive Treatment

**Figure 4:**
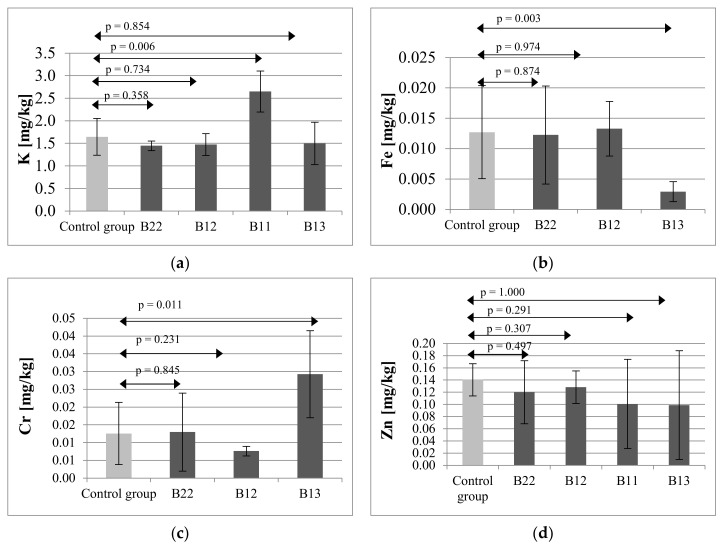

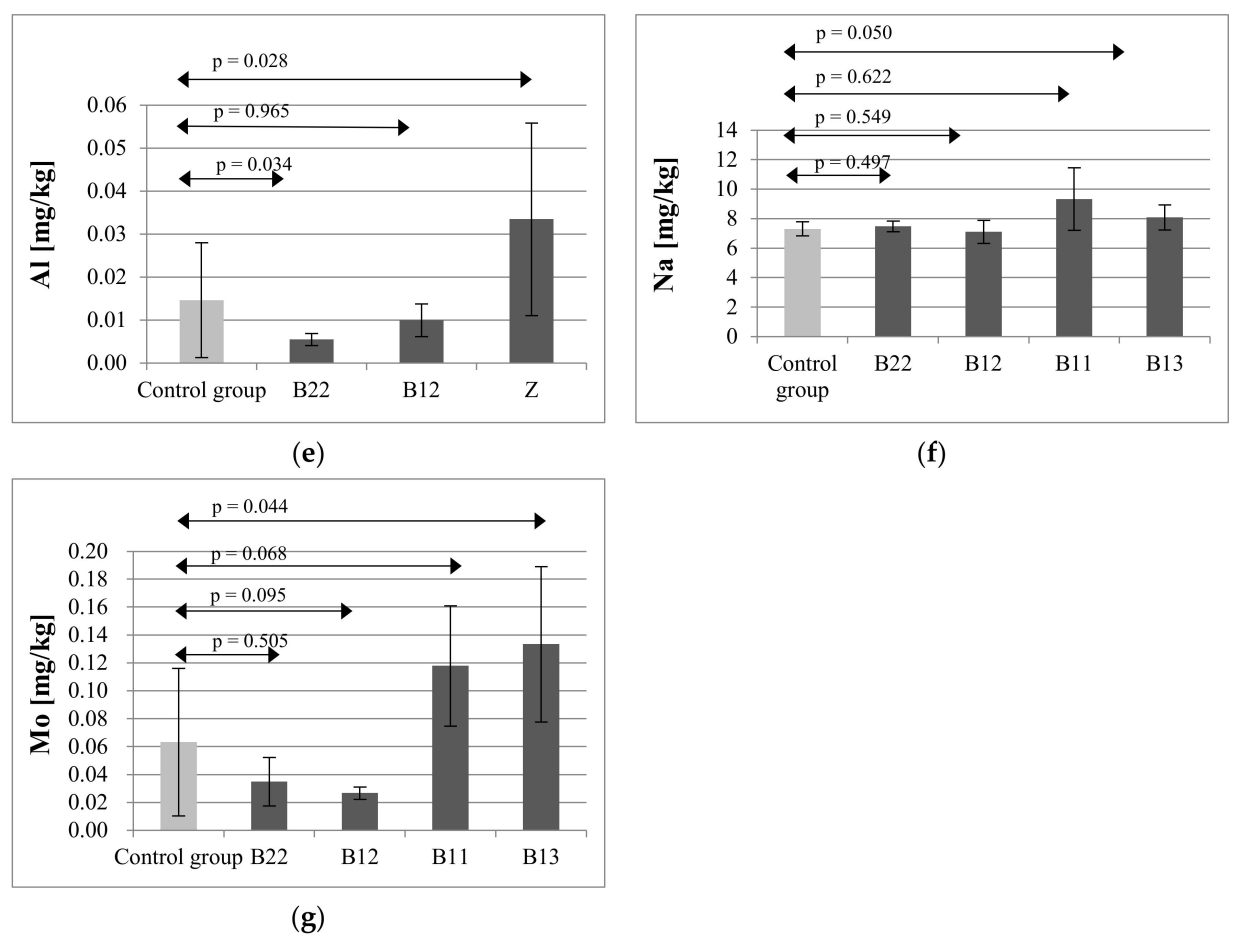
Mean concentration and SD of potassium (**a**), iron (**b**), chromium (**c**), zinc (**d**), aluminum (**e**), sodium (**f**) and molybdenum (**g**) in teeth of offspring of rat’s mothers receiving immunosuppressive therapy. B22—offspring of rat’s mothers receiving CsA (5 mg/kg/day), MMF (20 mg/kg/day), prednisone (4 mg/kg/day); B12—offspring of rat’s mothers receiving Tc (2 mg/kg/day), MMF (10 mg/kg/day), prednisone (4 mg/kg/day); B11—rat’s mothers receiving CsA (2.5 mg/kg/day), MMF (10 mg/kg/day), prednisone (4 mg/kg/day); B13—rat’s mothers receiving CsA (2.5 mg/kg/day), everolimus (0.25 mg/kg/day), prednisone (4 mg/kg/day). Statistically significant differences—*p* ≤ 0.05.

Analysis of dental tissues for potassium levels in the offspring of mothers receiving immunosuppressive therapies showed that the highest mean potassium level, amounting to 2.6502 mg/kg (SD = 0.4539), was found in the teeth of offspring in group B. The potassium level in group B22 amounted to 1.4475 mg/kg (SD = 0.1074), in group B12:1.4761 mg/kg (SD = 0.2424), and in group B13:1.4993 mg/kg (SD = 0.4710). Statistical analysis revealed a statistically significant difference between the control group and group B11 (*p* = 0.006) (Figure 4a).

The lowest iron level in teeth of the offspring exposed to immunosuppressants was found in group B13 (0.0029 mg/kg; SD = 0.0017), and it was significantly lower than the level in the control group of 0.0127 mg/kg (SD = 0.0077) (*p* = 0.003). In group B11, the iron content was below the detection limit LOD < 0.0009 (Figure 4b).

The mean content of chromium in teeth of the offspring in the control group amounted to 0.0126 mg/kg (SD = 0.0088), in group B22:0.0130 mg/kg (SD = 0.0110), in group B12:0.0076 mg/kg (SD = 0.0014), and in group B13:0.0293 mg/kg (SD = 0.0123). In the case of this mineral, statistical analysis revealed a significant difference between the control and group B13 (*p* = 0.011). The Cr content in group B11 was below the detection limit LOD < 0.0008 (Figure 4c).

Zinc content in the control group amounted to 0.1405 mg/kg (SD = 0.0265), in group B22:0.1201 mg/kg (SD = 0.0517), in group B12:0.1284 mg/kg (SD = 0.0268), in group B11:0.1008 mg/kg (SD = 0.0733), and in group B13:0.0989 mg/kg (SD = 0.0892). Due to the high standard deviations, statistical analysis did not show any significant differences between the groups: B22 (*p* = 0.497), B12 (*p* = 0.307), B11 (*p* = 0.291), B13 (*p* = 1.000) (Figure 4d).

In the analysis of aluminum concentrations in the teeth of the offspring exposed to immunosuppressive therapies, the highest content of this mineral, amounting to 0.0335 mg/kg (SD = 0.0224), was observed in group B. The Al level in group B22 amounted to 0.0055 mg/kg (SD = 0.0014), and in group B12:0.0100 mg/kg (SD = 0.0038). The level of this mineral in group B11 was below the detection limit LOD < 0.001. Statistical analysis revealed significant differences between the control and groups B22 (*p* = 0.034) and B13 (*p* = 0.028) (Figure 4e).

Sodium content in the control group and all the studied groups was on the similar levels therefore no statistically significant differences between the analyzed groups were identified for this mineral: B22 (*p* = 0.497), B12 (*p* = 0.549), B11 (*p* = 0.622), B13 (*p* = 0.050) (Figure 4f).

The highest Mo level in rat teeth was found in group B13 (0.1334 mg/kg; SD = 0.0557), and it was significantly higher than the level in the control group of 0.0634 mg/kg (SD = 0.0529) (*p* = 0.044) (Figure 4g).

## 3. Discussion

The use of combination therapy makes it possible to devise treatment regimens reducing the negative effects of immunosuppressive drugs causing bone resorption, but there is not much information on the effects of combination immunosuppressive therapies on mineral metabolism in human hard tissues [4,8,9,10]. It is known that bone metabolism is different in rats and humans; therefore, the effects of immunosuppressive drugs may be different. On the other hand, due to the toxic effects of immunosuppressive drugs on fetus such as MMF or mTOR inhibitors, these drugs can be examined only in an animal model [4].

Therefore, in this study, an attempt was undertaken to evaluate the effects of immunosuppression on the levels of potassium, iron, chromium, zinc, aluminum, sodium and molybdenum in hard tissues of female rats and their offspring.

Zinc is a cofactor for many metalloenzymes and proteins, and therefore it is involved in the regulation of bone cell function. The mineral stimulates cell differentiation, proliferation and mineralization by influencing gene expression of different proteins, including collagen type I, alkaline phosphatase and osteocalcin. It moreover inhibits bone resorption by inhibiting RANKL (receptor activator of nuclear factor κ B ligand) in pre-osteoclasts and stimulating OPG (osteoprotegerin) gene expression in osteoblasts [11]. Zn chelates to β-alanyl-L-histidine, forming AHZ. The process has a stronger stimulatory effect on bone formation than other osteogenetic factors [12]. A study by Samachson et al. demonstrated that the mineral part of bone contains more zinc than the part of the bone with poor mineralization [13]. In turn, Haumout reported that zinc concentration increases at the sites of bone calcification. By analogy, it has been suggested that similar Zn-modulated mechanisms occur in the hard tissues of teeth [14]. A study of children after liver transplantation showed that CsA and tacrolimus may cause dental defects from enamel opacities to hypoplasia, along with higher rates of tooth decay [15]. Furthermore, a study by Rahnama M. et al. revealed that corticosteroids cause a decrease in zinc concentrations in teeth [12]. In our study, it was observed that a regimen based on Tc, MMF and prednisone significantly reduces the zinc level in the bones of the offspring and teeth of the mothers, while a therapy featuring CsA, everolimus and prednisone significantly reduces the level of this mineral in teeth. It must be noted, however, that all therapies included in the study caused a decline in zinc content in the teeth of mothers and offspring, potentially compromising tooth development and strength. Like zinc, molybdenum acts as a cofactor for bone enzymes [16,17]. Out of the regimens included in the study, the greatest impact on Mo levels in bone and teeth was observed with the therapy based on CsA, prednisone and everolimus. Animal experiments revealed that molybdenum deficiency inhibits growth, particularly at the early stages of development [16]. On the other hand, studies investigating high molybdenum toxicity produced similar findings, including inhibition of fetal development, growth retardation and skeletal deformities [18,19].

To the best of our knowledge, the main functions of iron are oxygen transport and participation in many enzymatic systems in the body. These functions have a significant impact on normal bone homeostasis. Anemic hypoxia, which may ensue from iron deficiency, is a major stimulator of bone resorption, and besides Fe features as an essential cofactor for the hydroxylation of prolyl and lysyl residues of procollagen (lysyl oxidase) and antioxidant enzymes, removing oxygen free radicals produced in bone cells. Fe is also involved in vitamin D metabolism via cytochrome P450 mediation, consequently affecting the absorption of calcium [6,7]. Iron-deficiency anemia is the most common form of anemia after organ transplantation and may be caused by the use of immunosuppressive agents [20]. In our study, all therapies resulted in a decline of iron content in bone, which may potentially be due to anemia among the studied rats. On the other hand, the offspring exposed to full doses of CsA, MMF and prednisone presented with a significant increase in Fe levels. Please note that excessive exposure to this mineral may also contribute to the development of osteoporosis, upregulating bone resorption and oxidative stress and impairing biomechanical properties of bone [20]. Fe levels in teeth were significantly affected by the regimen based on CsA, everolimus and prednisone. In mother rats, the mineral content in teeth was maintained, possibly due to the fact that the therapy was introduced at a time when the teeth were fully formed and mineralized, but the offspring showed a significant decline.

Hard tissue demineralization accompanying the use of immunosuppressants may occur due to their direct toxic effect on kidneys. A reduced number of functional nephrons causes an overall impairment of processes taking place in renal tubules, leading to the so-called renal tubular acidosis (RTA) and increasing resorption of hard tissues. Metabolic acidosis may also result from a defect in proximal tubule ammonia synthesis, which may be caused by insulin resistance. Insulin normally induces ammonia synthesis in tubules, but it may be ineffective if immunosuppressive agents cause insulin resistance in transplant recipients [21].

Potassium is one of the minerals regulating the body’s pH balance. It promotes an alkaline environment, reducing the body’s need for salts building hard tissues (e.g., calcium salts, magnesium salts) to balance endogenous acid production due to the use of immunosuppressive medications so that calcium which would otherwise be mobilized to maintain normal pH can remain in hard tissues [21,22]. Research has shown a positive association between potassium intake and increased BMD in perimenopausal women, as well as hip and forearm BMD in older people, while on the other hand, low potassium intake increases bone resorption [6]. In our study, we noted that the analyzed immunosuppressive therapies affected potassium levels in hard tissues, which may be an effect of a disturbed acid–base balance and the resulting changes in bone metabolism.

Sodium is the extracellular counterpart of potassium. While it is believed that bone diseases are not related to sodium deficiency or overload, several studies demonstrated an elevated risk of osteoporosis and a higher prevalence of falls and fractures in relation to hyponatremia [23]. Hyponatremia has a direct effect on bone metabolism. Evidence shows that hyponatremia may stimulate osteoclast proliferation and activity, probably in order to mobilize the sodium stored in bone [23]. Seen as hyponatremia exacerbates osteoporosis, diets with a high salt content are recommended, but on the other hand, a high-sodium diet is also known to be harmful; hence sodium intake by individuals at risk or affected by osteoporosis (including those after immunosuppression) should be carefully monitored. The studied immunosuppressive regimens did not show a significant effect on mobilizing this mineral in bone or teeth [23].

Aluminum is an abundant element with a negative impact on bone health. It has an adverse effect on collagen synthesis, inhibiting bone formation and remodeling, which may lead to adynamic bone disease, renal osteodystrophy and osteomalacia [23]. Aluminum-induced osteomalacia is a form of renal osteodystrophy related to aluminum toxicity and its deposition in bone tissue, leading to disruptions in bone mineralization, cytotoxic effects on osteoblasts and aluminum accumulation in parathyroid cells [24]. In a study by Massari, a renal transplant recipient subjected to immunosuppressive treatment with steroids and cyclosporine A presented with increased aluminum concentrations in bone even 18 months post-transplantation [2]. While in our study, all groups of female rats had lower levels of this mineral than the control, in their offspring, the aluminum levels in bone were significantly elevated across all the groups, which may induce changes at the site of mineralization, affecting bone development [23].

As in the case of aluminum, high concentrations of chromium, too, have a toxic effect on bone cells, decreasing bone formation and inducing osteolysis. In rat studies, where pregnant rats were given drinking water with added chromium, females delivered pups with lower rates of mineralization in bone tissues [23,25]. In the case of therapy based on CsA, everolimus and prednisone, mother rats presented with lower levels of Al and Cr in teeth compared to the control group, while that in their offspring was significantly higher, indicating that the second generation in this group is particularly exposed to the toxic effects of aluminum and chromium [23].

In conclusion, the small number or lack of offspring in the groups exposed to full doses of immunosuppressive medications evidence the toxic effects of the drugs used and therapy regimens on rat fetuses. The immunosuppressive regimens under study affected the levels of minerals in the bones and teeth of the female rats and their offspring. The therapy based on CsA, everolimus and prednisone led to a decline in the levels of iron in bone, zinc in teeth, and molybdenum in the bone and teeth of mothers, while in the offspring, it caused a decline of bone potassium, with a decrease in iron and increase of molybdenum in teeth. While all therapies increased the aluminum level in bone for the offspring, the regimen based on CsA, everolimus and prednisone additionally caused an increase in aluminum and chromium in the teeth of the offspring, and consequently, it seems to be the therapy with the most negative impact on the mineral metabolism in hard tissues.

## 4. Material and Methods

### 4.1. Characteristics of the Study Population

The study was conducted on 32 female Wistar rats at twelve weeks of age, with mean body weight at baseline amounting to 230 g. The animals were acquired from the Experimental Medicine Center of the Medical University of Białystok (Białystok, Poland), and the study was approved by the Local Animal Research Ethics Committee in Szczecin (no 12/2013, dated 24 October 2013). Upon arrival, rats were kept in single cells with a 12 h light–dark cycle and fed Labofeed H (Morawski, Kcynia, Poland) and water ad libitum. Females participating in the study were divided into four groups—the control group (C) and three study groups, (B) receiving different drug combinations. Each group consisted of eight female rats (*n* = 8). Medications were administered by oral gavage. Dosages were based on literature data [26,27,28,29,30,31,32,33,34]. Rats from the control group were given carrier and olive oil, while rats in experimental groups were given medications in a pharmaceutical form so as to obtain body concentrations in the therapeutic range. The immunosuppressive therapy regimens used in the study groups are presented below:

Group B1 (*n* = 8)
  - prednisone (Encorton, Polfa, Warsaw, Poland): 4 mg/kg/day
  - CsA (Sandimmun Neoral, Novartis, Basel, Switzerland): 5 mg/kg/day
  - MMF (CellCept; Hoffmann-La Roche Ltd., Basel, Switzerland): 20 mg/kg/day
Group B2 (*n* = 8)
  - prednisone (Encorton, Polfa, Warsaw, Poland): 4 mg/kg/day
  - MMF (CellCept; Hoffmann-La Roche Ltd., Basel, Switzerland): 20 mg/kg/day
  - Tc (Prograf, Astellas, Northbrook, IL, USA): 4 mg/kg/day
Group B3 (*n* = 8)
  - prednisone (Encorton, Polfa, Warsaw, Poland): 4 mg/kg/day
  - CsA (Sandimmun Neoral, Novartis, Basel, Switzerland): 5 mg/kg/day
  - everolimus (Certican; Novartis, Basel, Switzerland): 0.5 mg/kg/day

The animals received medication every 24 h for approximately 5 weeks (2 weeks after the acclimatization period prior to mating—when placed with males 1:1 in separate cages—and later after mating during 3 weeks of pregnancy). Once a week, the animals were weighed, and the drug dosage was adjusted to the current weight. After delivery, the treatment was stopped (no drug administration during the lactation period). Thirty-one female rats completed the study. Sixty-nine pups were born in the control group, 13 pups in group B1 (with the offspring of group B1 identified as group B22) and one pup in group B3.

The number of pups born in the study groups was small, and therefore the experiment was repeated with medication dosage reduced by half, except for the dose of the corticosteroid, which remained the same. The study was conducted on eight female rats at 12 weeks of age, divided into three groups. The experiment was approved by the Local Animal Research Ethics Committee in Szczecin (no 10/2014 and 11/2014, both dated 06 June 2014). The animals from the study groups were given medications according to three regimens:

Group B11 (*n* = 2)
  - prednisone (Encorton, Polfa, Warsaw, Poland): 4 mg/kg/day
  - CsA (Sandimmun Neoral, Novartis, Basel, Switzerland): 2.5 mg/kg/day
  - MMF (CellCept; Hoffmann-La Roche Ltd., Basel, Switzerland): 10 mg/kg/day
Group B12 (*n* = 3)
  - Tc (Prograf, Astellas, Northbrook, IL, USA): 2 mg/kg/day
  - MMF (CellCept; Hoffmann-La Roche Ltd., Basel, Switzerland): 10 mg/kg/day
  - prednisone (Encorton, Polfa, Warsaw, Poland): 4 mg/kg/day
Group B13 (*n* = 3)
  - CsA (Sandimmun Neoral, Novartis, Basel, Switzerland): 2.5 mg/kg/day
  - everolimus (Certican; Novartis, Basel, Switzerland): 0.25 mg/kg/day
  - prednisone (Encorton, Polfa, Warsaw, Poland): 4 mg/kg/day.

To evaluate drug concentrations in the rats’ blood, we used a separate group of female rats (*n* = 14) of the same age that were pregnant. The concentration of CsA was determined with the Abbott AxSYM assay, which is based on fluorescence. To determine the Tc level, we used the IMx assay based on microparticle enzyme immunoassay. The concentration of everolimus was determined at the Laboratory of Mass Spectrometry.

### 4.2. Sample Preparation and Decomposition

The rats were euthanized at the age of 19 days and 8 weeks by penthobarbitalum sodium injection administered intraperitoneally at 40 mg/kg body weight. Their hard tissues were collected for further study. Rat bone and tooth material collected during necropsy was dried at 100 °C until dry mass was obtained. Dry tissue was crushed with an agate mortar and pestle, and 100 mg samples were weighed into plastic vials and labeled. After preparation, the samples were subjected to a microwave decomposition procedure using a microwave digestion system. To this end, 2 mL of 65% HNO3 was poured on top of the 100 mg samples, transferred into Teflon vessels and placed in the microwave digester. The digestion process was divided into two stages—the first lasting 15 min, during which the samples were gradually heated up to 180 °C, and the second, lasting 20 min, during which the temperature was maintained at 180 °C.

### 4.3. Elemental Composition Analysis

Elemental analysis was carried out using inductively coupled plasma optical emission spectrometry (ICP-OES, ICAP 7400 Duo, Thermo Scientific, Waltham, MA, USA). The digested samples were diluted 20-fold, 500 µL yttrium was added as the internal standard at 0.5 mg/L and 1 mL of 1% Triton (Triton X-100, Sigma-Aldrich, Poznan, Poland). The samples were diluted with 0.075% HNO_3_ (Suprapur, Merck, Poznan, Poland) up to the volume of 10 mL and stored in the fridge until analysis.

The calibration curve was constructed using multielement standard solutions (ICP multielement standard solution IV, IX and XVI, Merck), which were prepared with deionized water (Direct Q UV, Millipore, approx.18.0 MΩ).

## 4.4. Statistical Analysis

Data analysis was carried out using the Statistica 13 software package (TIBCO Software Inc., Palo Alto, California, USA). Data distribution deviated from a normal distribution, and therefore a nonparametric Mann–Whitney U test was used in further analysis of statistical significance. The threshold of statistical significance was set at *p* ≤ 0.05.
